# Draft genome sequence of plant growth-promoting bacterial endophyte *Cellulosimicrobium cellulans* strain MUKR 5 isolated from *Iris laevigata* Fisch. from Manipur, India

**DOI:** 10.1128/mra.00892-25

**Published:** 2025-12-23

**Authors:** Shantirani Thokchom, Saikat Mukherjee, Debananda Singh Ningthoujam

**Affiliations:** 1 Department of Biochemistry, Manipur University 29661, Imphal, Manipur, India; California State University San Marcos, San Marcos, California, USA

**Keywords:** Indo-Myanmar Biodiversity hot spot, ethomedicinal plant, endophytic bacteria, whole-genome sequencing, plant growth promotion, biosynthetic gene cluster

## Abstract

We report a plant growth-promoting bacterial endophyte, *Cellulosimicrobium cellulans* MUKR 5, isolated from *Iris laevigata* Fisch., an ethnomedicinal plant belonging to Manipur, a North-Eastern State of India, in the Indo-Myanmar Biodiversity hot spot. The draft genome consists of 4,229,506 bp (GC 74.5%) assembled into three contigs.

## ANNOUNCEMENT

*Iris laevigata* Fisch. (water iris), a rhizomatous perennial from East Asia (1), recently documented from Manipur, India, belongs to the Indo-Myanmar Biodiversity hot spot (2). Known locally as Kombirei, its rhizomes are used as a brain coolant and for treating hysteria (3). Ethnomedicinal plants host bioactive endophytes (4). From *I. laevigata* roots, *Cellulosimicrobium cellulans* strain MUKR 5 was isolated. Plant roots collected from Mayang Imphal, Manipur (24°62′11″N, 93°87′97″E) underwent five-step surface sterilization (sequential washes with NaOCl, Na_2_S_2_O_3_, C_2_H_5_OH, H_2_O, and NaHCO_3_), followed by triple sterile distilled water (SDW) rinses (5). Sterile roots were cut and plated on starch casein nitrate agar, supplemented with 100 μg/mL of cycloheximide (antifungal) and nalidixic acid (antibiotic), incubated at 30°C for 3–4 weeks until morphologically discreet bacterial colonies emerged, followed by subculturing twice at 30°C for 5 days onto fresh plates to obtain pure culture. Taxonomic identification via 16S rRNA gene sequencing involved genomic DNA extraction (Xploregen Universal gDNA Kit), PCR amplification (forward primer: GGATGAGCCCGCGGCCTA and reverse primer: CGGTGTGTACAAGGCCCGG), purification (QIAquick PCR Purification Kit), and sequencing (BigDye Terminator v3, ABI 3130xl Genetic Analyzer). SeqScape v5.2 and BLAST (vBLAST+2.16.0) analysis revealed 100% similarity to *C. cellulans* NEB113 (accession no. CP041694.1) in NCBI RefSeq (Release 229). Phylogeny was constructed in MEGA v11 (6) using the neighbor-joining method.

Bacteria were cultured at 30°C in nutrient broth (NB). DNA was extracted (Xploregen Universal gDNA Kit), quantified (100 ng) (Qubit 3.0 Fluorometer, dsDNA HS dye), and fragmented to 200–300 bp. A genomic DNA library was prepared using the NEBNext Ultra II DNA Library Prep Kit for Illumina (New England Biolabs) following the manufacturer’s protocol. Fragments underwent end repair, 3′ adenylation, loop adapter ligation, and USER enzyme (NEB) cleavage. Libraries were purified (AMPure beads), PCR-enriched (six cycles, NEBNext Ultra II Q5 master mix with Illumina primers), cleaned, and eluted in 0.1× TE. Libraries were quantified (Qubit) and analyzed (Agilent 2100 Bioanalyzer, DNA 7500 chip). Sequencing (2 × 150 bp) on an Illumina NovaSeq 6000 yielded 8,964,178 reads (1.35 Gbp), with a depth of 261.08×, and a mean coverage of 99.95. Reads were quality-checked (fastp v10.1) (7), trimmed (Trim Galore v0.6.10) (8), assembled (Unicycler v0.5.1) (9), scaffolded (Multi-CSAR v1.0) (10), and the resulting contigs were visualized in a circular layout using CGView v2.0.3 (11) for representation purposes. Taxonomic identity was verified using pubMLST (database updated 13 March 2025) (12), and genome annotation was completed using the NCBI Prokaryotic Genome Annotation Pipeline v6.10. Assembly statistics are summarized in Table 1. Default parameters were used for the software, except where otherwise noted.

**TABLE 1:** Statistics of the genome assembly

Parameter	Value
Genome size	4.2 Mb
Total ungapped length	4.2 Mb
Number of scaffolds	3
Scaffold N50	4 Mb
Scaffold L50	1
No. of contigs	3
Contig N50	4 Mb
Contig L50	1
GC %	74.5
Genome coverage	99.95×
Assembly level	Contig

**Fig 1:**
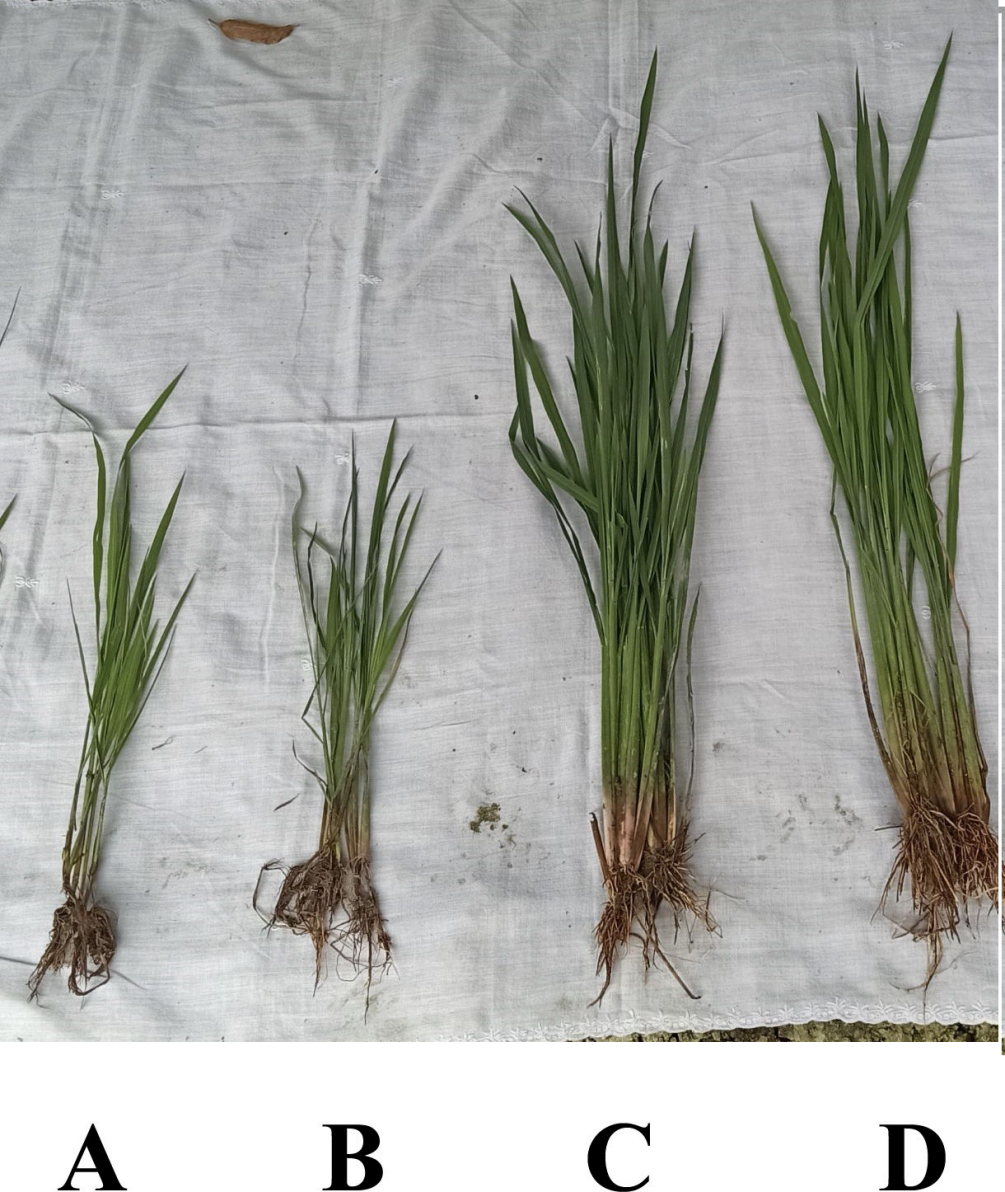
Plant growth promotion in rice seedlings under nethouse conditions with four treatments: (**A**) SDW control, (**B**) NB control, (**C**) urea control, and (**D**) MUKR 5.

The draft genome was assembled in three contigs (4,229,506 bp and GC 74.5%). A total of 3,832 genes were identified, including 3,727 protein-coding sequences, 57 RNA genes (one copy of the 16S and 23S rRNA genes, 52 tRNAs, and 3 ncRNAs), and 48 pseudogenes. Rice plants (var. Thoibi) grown in sterilized sandy-loam soil for 60 days in the presence of MUKR 5 in nethouse conditions showed significantly increased root length, shoot length, and biomass as compared to controls (Fig. 1).

## Data Availability

The following information is available in GenBank: 16S rRNA gene sequence, PV300543; whole-genome data, PRJNA1247247; assembly, GCF_049629635.1; WGS master, JBMYHP000000000; BioSample, SAMN47808382; and SRA, SRX28774837.
